# Effects of CTLA4-Ig on human monocytes

**DOI:** 10.1186/s41232-017-0054-5

**Published:** 2017-11-06

**Authors:** Toshihiro Tono, Satoko Aihara, Takayuki Hoshiyama, Yoshiyuki Arinuma, Tatsuo Nagai, Shunsei Hirohata

**Affiliations:** Department of Rheumatology and Infectious Diseases, Kitasato University School of Medicine, 1-15-1 Kitasato, Minami-ku, Sagamihara, Kanagawa 252-0374 Japan

**Keywords:** Abatacept, Monocytes, Apoptosis, Costimulation molecules

## Abstract

**Background:**

Abatacept, a CTLA4-Ig fusion protein attenuates T cell activation by inhibiting the CD80/86-CD28 costimulatory pathway that is required for the proper T cell activation and thus displays beneficial effects in the treatment of rheumatoid arthritis (RA). Although some studies have disclosed the in vitro effects of this biological agent on the immune-competent cells, the precise mechanisms of action in RA still remain unclear. The current studies were therefore undertaken to explore the effects of abatacept on monocytes in detail.

**Methods:**

Monocytes from healthy donors were cultured in the presence of staphylococcal enterotoxin B (SEB) with pharmacologically attainable concentrations of abatacept or control IgG-Fc. The expression of CD80 and CD86 and the induction of apoptosis of monocytes were measured by flow cytometry. The expression of CD80 and CD86 messenger RNA (mRNA) was determined by quantitative RT-PCR.

**Results:**

Abatacept promoted apoptosis of SEB-stimulated monocytes. The induction of apoptosis of monocytes by these biological agents was reversed by the addition of IgG, but not IgG-F(ab′)_2_ fragments. Furthermore, abatacept significantly suppressed the expression of CD80, but not that of CD86 at protein levels. Finally, abatacept significantly suppressed the expression of mRNA for CD80 of monocytes stimulated with SEB, but not that of CD86.

**Conclusions:**

These results demonstrate that one of the mechanisms of action of abatacept involves the induction of apoptosis of monocytes, which involves interaction with Fc receptor on monocytes. Moreover, the data also demonstrate that abatacept selectively suppresses the expression of CD80 at mRNA levels.

## Background

Abatacept, a CTLA4-Ig fusion protein, attenuates T cell activation by inhibiting the CD80/CD86–CD28 costimulatory pathway that is required for the proper T cell activation and thus displays beneficial effects in the treatment of rheumatoid arthritis (RA) [1]. Although some studies have disclosed the in vitro effects of this biological agent on the immune-competent cells [1], the precise mechanisms of action in RA still remain unclear.

CTLA4-Ig has been suggested to display some effects other than inhibition of T cells. Thus, reverse signaling to dendritic cells upon binding of CTLA4-Ig to CD80/CD86 has been shown to interfere with dendritic cell activation and function [2, 3]. Since monocytes express CD80/CD86 as well [4], it is possible that abatacept might influence the function of monocytes. The current studies were therefore undertaken to explore the effects of abatacept on monocytes in detail. Special attention was paid to the capacities of this biological agent to induce apoptosis of monocytes and to modulate the expression of costimulation molecules.

## Methods

### Monoclonal antibodies

A variety of monoclonal antibodies (mAbs) were used in this study, including fluorescein isothiocyanate (FITC)-conjugated anti-CD80 (Immunotech, Marseille, France), FITC-conjugated anti-CD86 (Ancell, Bayport, MN), and FITC-conjugated control mouse IgG1 (Dako, Glostrup, Denmark).

### Cell preparation

Peripheral blood mononuclear cells (PBMCs) were obtained from healthy adult volunteers who gave informed consent, by centrifugation of heparinized venous blood over sodium diatrizorate-Ficoll gradients. Monocytes were prepared from PBMC using Monocyte Isolation Kit II (Miltenyi Biotec). Monocyte population obtained in this manner contained < 0.1% CD3+ cells, < 0.1% CD19+ cells, and > 93% CD14+ cells.

### Reagents

Abatacept was purchased from Bristol-Myers Squibb (Tokyo). Control human IgG1 was purified from serum of a patient with human IgG1 myeloma using DEAE-Sepharose column. Human IgG-Fc and human IgG-F(ab′)_2_ (Gamma-Venin^®^ P) were purchased from MP Biomedicals, Santa Ana, CA, and Sanofi, Paris, France, respectively.

### Cell cultures

RPMI 1640 medium (Nikken, Kyoto, Japan) supplemented with penicillin G (100 U/ml) (Life Technologies, Grand Island, NY), streptomycin (100 μg/ml) (Life Technologies), l-glutamine (0.3 mg/ml) (Sigma-Aldrich, St Louis, MO), and 10% fetal bovine serum (JRH Bio Sciences, Lenexa, KS) were used for cultures. Purified monocytes (1 × 10^6^/well) were cultured in the presence of staphylococcal enterotoxin B (SEB) (100 pg/ml) (Serva, Heidelberg, Germany) in each well of 24-well flat-bottomed microtiter plates (Nunc, Roskilde, Denmark) with control IgG (100 μg/ml), IgG-Fc (100 μg/ml), or abatacept (100 μg/ml) for 24 or 48 h. SEB stimulation and the addition of abatacept or IgG were simultaneous. Also, purified monocytes (1 × 10^6^/well) were cultured with or without SEB (100 pg/ml) in each well of 24-well flat-bottomed microtiter plates (Nunc, Roskilde, Denmark) with or without IgG (100 μg/ml) or IgG-F(ab′)_2_ (100 μg/ml) with abatacept (100 μg/ml) for 48 h.

### Analysis of cell surface antigens by flow cytometry

The surface antigens on monocytes and the induction of apoptosis were analyzed by flow cytometry. Briefly, after the incubation for 48 h, the cells were washed once by phosphate-buffered saline (PBS) containing 2% normal human serum and 0.1% sodium azide (staining buffer). The cells were then reacted in suspension with saturating concentrations of FITC-conjugated mAbs for 30 min at 4 °C. After the cells were washed once with staining buffer and then twice with PBS, they were counterstained with phycoerythrin (PE)-conjugated annexin V (R&D Systems, Minneapolis, MN), according to the manufacturer’s instructions. The cells were then analyzed using Cell Lab Quanta SC (Beckman Coulter, Miami, FL). To identify viable cells, the gating for the staining with annexin V was used. In some experiments, the cells were stained with FITC-conjugated annexin V (R&D Systems) and propidium iodide (PI) and were analyzed by flow cytometry.

### RNA isolation and real-time quantitative PCR

Total RNA was isolated from cultured cells using ISOGEN (Nippon Gene, Tokyo, Japan) according to the manufacturer’s specifications. cDNA was prepared from 1 μg of total RNA using M-MLV reverse transcriptase (Takara Bio, Shiga, Japan) with random primers (Takara Bio) and was subjected to analysis with real-time PCR using LightCycler 4.1 (Roche Diagnostics, Lewes, UK). Real-time PCR of CD80, CD86, and β-actin was performed using SYBR Premix Ex Taq II (Takara Bio) with the following primers: sense, 5′-AATGCACATCTCATGGCAGCTAA-3′, and antisense, 5′-AGGTTTGTGAAGCAGCATAGTGAGG-3′, for CD80 (NM-005191.3); sense, 5′-TGGCCTAGGGTACAGGCAACA-3′, and antisense, 5′-GCCCAGATAGAAGTGGCTCCAG-3′, for CD86 (NM-001206924.1); and sense, 5′-TGGCACCCAGCACAATGAA-3′, and antisense, 5′-CTAAGTCATAGTCCGCCTAGAAGCA-3′, for β-actin (NM-000600). Amplification was performed according to the standard protocol recommended by the manufacturer. All results were calibrated to the copy number of β-actin obtained from the same cDNA samples.

### Statistical analysis

Statistical significance was evaluated by Wilcoxon’s signed-rank test.

## Results

### Effects of abatacept on annexin V expression of SEB-stimulated monocytes

**Fig. 1:**
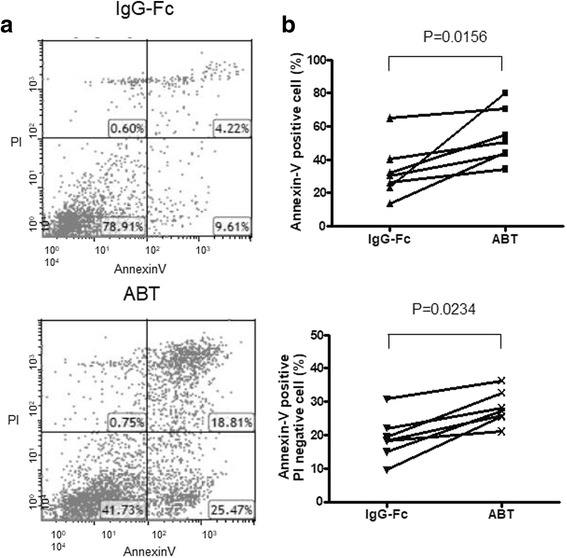
Effects of abatacept on annexin V expression of SEB-stimulated monocytes. Highly purified monocytes (1 × 10^6^/well) from eight healthy individuals were cultured in the presence of SEB (100 pg/ml) in each well of 24-well flat-bottomed microtiter plates with abatacept (100 μg/ml) or control IgG-Fc (100 μg/ml) for 48 h, after which the cells were stained with FITC-conjugated annexin V and propidium iodide (PI). The cells were then analyzed by flow cytometry. **a** Representative dot plots for staining with FITC-conjugated annexin V and PI. **b** Percentages of total annexin V-positive cells (upper) or PI-negative annexin V-positive cells (lower) are shown. Statistical significance was evaluated by Wilcoxon’s signed-rank test

Initial experiments examined the effects of abatacept on annexin V expression of SEB-stimulated monocytes to explore their influences on the induction of apoptosis. As shown in Fig. 1, abatacept increased the expression of annexin V of monocytes compared with control IgG-Fc. It should be noted that abatacept also increased the numbers of annexin V-positive and PI-negative cells. The results therefore indicate that abatacept promotes apoptosis of SEB-stimulated monocytes. Moreover, the data also confirm that human monocytes are direct targets of abatacept.

**Fig. 2:**
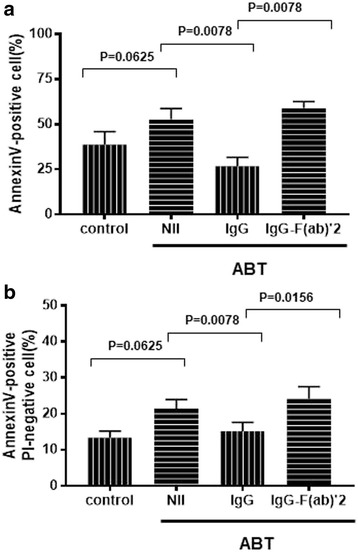
Differential effects of human IgG and IgG-F(ab′)_2_ fragments on the induction of apoptosis of SEB-stimulated monocytes by abatacept. Highly purified monocytes (1 × 10^6^/well) from healthy individuals were cultured in the presence of SEB (100 pg/ml) in each well of 24-well flat-bottomed microtiter plates with abatacept (100 μg/ml) for 48 h, with or without IgG (100 μg/ml), or with IgG-F(ab′)_2_ fragments (100 μg/ml). After the cultures, the cells were stained with PE-conjugated annexin V, followed by analysis on flow cytometry. Percentages of total annexin V-positive cells (upper, *n* = 3) or PI-negative annexin V-positive cells (lower, *n* = 3) are shown. Error bars indicate SD values of three different experiments. Statistical significance was evaluated by Wilcoxon’s signed-rank test. V-positive cells (**a**) and PI-negative annexin V-positive cells (**b**)

It was previously disclosed that etanercept was able to induce apoptosis of TNF-α expressing Jurkat T cells only in the presence of human PBMCs, whereas infliximab was able to induce their apoptosis through outside to inside signal via TNF-α in the absence of PBMCs [5]. It is therefore possible that interactions with Fc receptors on monocytes might be required for the induction of apoptosis of monocytes by these biological agents. We next examined the influences of IgG and IgG-F(ab′)_2_ fragments on the capacities of abatacept to induce apoptosis of SEB-stimulated monocytes. As shown in Fig. 2, the addition of IgG, but not IgG-F(ab′)_2_, almost completely reversed the capacities of abatacept to induce apoptosis of SEB-stimulated monocytes. These results indicate that intact IgG rather protects monocytes from apoptosis. More importantly, the data demonstrate that Fc receptors on monocytes are involved in the induction of apoptosis of SEB-stimulated monocytes by abatacept. Also, we examined these experiments without SEB stimulation. Although the results of the experiments of apoptosis were similar to those with SEB stimulation, they did not reach the statistically significant difference.

### Effects of abatacept on the expression of CD80 and CD86 on SEB-stimulated monocytes

**Fig. 3:**
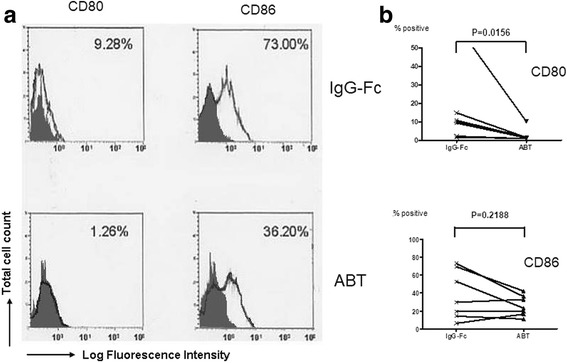
Effects of abatacept on the expression of CD80 and CD86 on SEB-stimulated monocytes. Highly purified monocytes (1 × 10^6^/well) from seven healthy individuals were cultured in the presence of SEB (100 pg/ml) in each well of 24-well flat-bottomed microtiter plates with abatacept (100 μg/ml) or control IgG-Fc (100 μg/ml) for 48 h, after which the cells were stained with FITC-conjugated anti-CD80, anti-CD86, or control IgG1, followed by counterstaining with PE-conjugated annexin V. The cells were then analyzed by flow cytometry. **a** Representative histograms of the staining of various molecules on annexin V-negative monocytes. The percentages of positive cells for specific mAb staining are indicated. Stainings with isotype-matched control mAb (control IgG1) are indicated by shade. **b** Percentages of positive cells for each specific mAb staining of monocytes from seven independent experiments are summarized. Statistical significance was evaluated by Wilcoxon’s signed-rank test

The next experiments examined the effects of abatacept on the expression of costimulatory molecules on monocytes stimulated with SEB. Figure 3a shows the typical histogram of the expression of CD80 and CD86 on annexin V-negative monocytes from a normal healthy donor. Abatacept suppressed the expression of CD80, but not as much as the expression of CD86 on annexin V-negative monocytes from a normal healthy donor. Accordingly, abatacept significantly inhibited the expression of CD80, but not that of CD86, on SEB-stimulated monocytes compared with control IgG-Fc in seven healthy individuals (Fig. 3b). The results indicate that the expression of CD80 and that of CD86 on monocytes are regulated in different mechanisms in such that CTLA4-Ig suppresses the former alone.

**Fig. 4:**
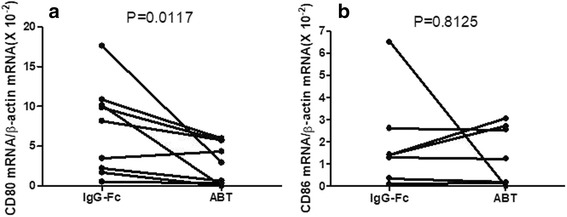
Effects of abatacept on the mRNA expression for CD80 and CD86 of SEB-stimulated monocytes. Highly purified monocytes (1 × 10^6^/well) from healthy individuals were cultured in the presence of SEB (100 pg/ml) in each well of 24-well flat-bottomed microtiter plates with abatacept (100 μg/ml) or control IgG-Fc (100 μg/ml). After 24 h of incubation, the cells were harvested and the expression of mRNA for CD80 and CD86 were examined by quantitative RT-PCR. Data are expressed as the ratio to copy numbers of β-actin mRNA. Statistical significance was evaluated by Wilcoxon’s signed-rank test. CD80 (**a**) and CD86 (**b**)

Next, experiments examined the effects of abatacept on the expression of messenger RNA (mRNA) for CD80 and CD86 of SEB-stimulated monocytes. As shown in Fig. 4, abatacept significantly suppressed the expression of CD80 mRNA in SEB-stimulated monocytes but not that of CD86 mRNA compared with control IgG-Fc in seven healthy individuals. The results confirm that abatacept has direct effects on monocytes to suppress their expression of CD80 at mRNA levels. Moreover, the data suggest that the expression of CD80 mRNA and CD86 mRNA might be regulated in different mechanisms.

## Discussion

The current studies clearly demonstrate that abatacept not only increased annexin V-positive cells which represent cell death but also increased annexin V-positive and PI-negative cells representing apoptosis in this system. The results therefore indicate that abatacept induces apoptosis of SEB-stimulated monocytes. Since the addition of normal human IgG, but not IgG-F(ab′)_2_ fragments, inhibited the induction of apoptosis by abatacept, it is suggested that Fc receptors on monocytes are involved in the induction of apoptosis of SEB-stimulated monocytes by abatacept. Previous studies showed that antibody-dependent cell cytotoxicity (ADCC) is one of the mechanisms of induction of apoptosis in target cells, in which Fc receptors on effector cells play a pivotal role [6]. Since the addition of intact IgG almost completely abrogated the capacity of abatacept to induce apoptosis of SEB-stimulated monocytes in the present study, it is suggested that induction of apoptosis of human monocytes by this biological compound might be mediated through ADCC. Thus, the data suggest the possibility that the co-cross-linking of CD80/CD86 and Fc receptors by CTLA4-Ig might result in the promotion of apoptosis of the SEB-stimulated monocytes. However, in a recent study, it was reported that the Fc region of abatacept does not bind to CD16 (FcγRIII) and CD32 (FcγRII) and does so weakly to the CD64 (FcγRI) of B cells [7]. To confirm the role of Fc receptors in the apoptosis observed in this system, experiments using anti-Fcγ (FcγRIII, FcγRII, and FcγRI) receptor-neutralizing antibody would be important. Since the Fc region of abatacept binds only weakly to the FcγRI, it is possible that abatacept results in the apoptosis of monocytes in a mechanism other than ADCC. It should be emphasized that a number of signals may cause apoptosis. Therefore, it is possible that co-cross-linking of CD80/CD86 and Fc receptors by abatacept might deliver such signals that lead to apoptosis of monocytes. Further studies to explore such signals would be important for our complete understanding of the mechanism of action of abatacept.

It was found that etanercept and infliximab induced apoptosis of macrophages/monocytes in the synovial fluid in vitro [8]. Although abatacept has been shown to increase the apoptosis of CD4-positive T cells by blocking of costimulation signals in the synovial fluid of RA patients [9], it has been unclear whether abatacept induces apoptosis of monocytes in vivo. It should be pointed out that SEB activates monocytes and dendritic cells through Toll-like receptor (TLR) 2 [10]. On the other hand, previous studies demonstrated that the expression of TLR2 was upregulated in peripheral blood monocytes, synovial macrophages, and synovial tissue of RA, thus playing an important role in the pathogenesis [11, 12]. In the present study, we have demonstrated that abatacept increased the number of annexin V-positive and PI-negative monocytes, presumably cells in the early phase of apoptosis. It is, therefore, strongly suggested that abatacept might also induce apoptosis of monocytes in vivo in RA. In addition, since macrophages/monocytes derived from the bone marrow are precursors of type A synoviocytes [6, 13], it is also likely that abatacept might cause apoptosis of type A synoviocytes as well as lead to the reduction of recruitment of monocytes/macrophages into the synovium. Taken together, the results confirm that one of the important mechanisms of action of abatacept involves the induction of apoptosis of activated monocytes/macrophages.

While the binding of CD28 to CD80 or CD86 promotes the full activation of a T cell receptor (TCR)-stimulated T cell, the cross-linking of CTLA4 concomitant with TCR triggering results in the inhibition of proliferation and cytokine secretion [9]. Thus, inhibition of T cell activation is considered to be one of the critical mechanisms of action of abatacept [9]. It is well known that bacterial superantigens, including SEB, induce the expression of CD80 on monocytes [10, 14]. The current studies demonstrate that the expression of CD80, but not that of CD86, was significantly inhibited by abatacept at mRNA levels. The downregulation of CD80 on monocytes by abatacept might further facilitate the inhibitory effects of T cell activation.

The precise sequelae of transcriptional regulation of CD80 and CD86 have not been delineated. Recently, Kaneda et al. reported the role of a transcription factor PU.1 in the regulation of CD80 and CD86 expression in dendritic cells (DCs) [15]. Thus, PU.1 is a transcription factor belonging to the ETS family and plays a key role in the gene expression of CD80 and CD86 in bone marrow-derived DCs. However, since abatacept did not suppress the expression of mRNA for CD86, it is suggested that some transcription factors other than PU.1 might play a role in the gene expression of CD80. Further studies to explore the mechanisms on the expression of mRNA for CD80 and CD86 would be necessary.

## Conclusions

The results in the current studies have delineated the influences of abatacept on human monocytes, possibly by ADCC, which involves interaction with Fc receptor on human monocytes. The characteristic features of this biological agent include the induction of apoptosis and the inhibition of the expression of CD80 mRNA, but not that of CD86 mRNA. Further studies to delineate the precise mechanisms of transcription of CD80 and CD86 would be important.

## Abbreviations

ADCC: Antibody-dependent cell cytotoxicity
CTLA4-Ig: Abatacept
DCs: Dendritic cells
FITC: Fluorescein isothiocyanate
PBMC: Peripheral blood mononuclear cell
PBS: Phosphate-buffered saline
PE: Phycoerythrin
PI: Propidium iodide
RA: Rheumatoid arthritis
SEB: Staphylococcal enterotoxin B
TCR: T cell receptor
TLR: Toll-like receptor
